# QuickStats

**Published:** 2013-02-08

**Authors:** Marian F. MacDorman, T.J. Mathews

**Figure:**
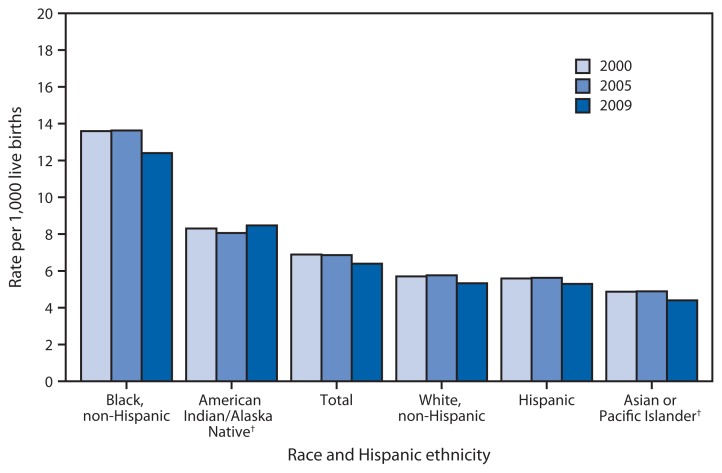
Infant Mortality Rates,* by Race and Hispanic Ethnicity of Mother — United States, 2000, 2005, and 2009 * Per 1,000 live births. ^†^ Includes persons of Hispanic and non-Hispanic ethnicity.

During 2000–2005, the U.S. infant mortality rate did not decline significantly for the total population or for any racial/ethnic population. However, from 2005 to 2009, the rate declined by 7% to 6.39 infant deaths per 1,000 live births and declined significantly for all racial/ethnic groups except for American Indian/Alaska Native women. Infant mortality rates in 2009 were higher than the U.S. average (6.39) for non-Hispanic black (12.40) and American Indian/Alaska Native women (8.47). Rates were lower than the U.S. average for non-Hispanic white (5.33), Hispanic (5.29) and Asian or Pacific Islander women (4.40).

**Source:** Mathews TJ, MacDorman MF. Infant mortality statistics from the 2009 period linked birth/infant death data set. Natl Vital Sat Rep 2012;61(8).

